# Quantitative Benefit-Risk Models Used for Rotavirus Vaccination: A Systematic Review

**DOI:** 10.1093/ofid/ofaa087

**Published:** 2020-03-12

**Authors:** Hugo Arlegui, Gaëlle Nachbaur, Nicolas Praet, Bernard Bégaud

**Affiliations:** 1 INSERM, Bordeaux Population Health Research Center, Team Pharmacoepidemiology, UMR 1219, University of Bordeaux, Bordeaux, France; 2 Pharmaco-Epidemiology and Health Outcomes Research, GSK, Rueil-Malmaison, France; 3 Clinical Research and Development, GSK, Wavre, Belgium

**Keywords:** benefit-risk, intussusception, rotavirus, vaccines and immunization, systematic review

## Abstract

**Background:**

Although rotavirus vaccines have proven to prevent the risk of rotavirus gastroenteritis (RVGE) in children under 5 years old, they are also associated with an increased transient risk of intussusception (IS). Several quantitative benefit-risk models (qBRm) are performed to measure this balance in hospitalizations and deaths prevented versus the ones induced.

**Method:**

In this study, our objective was to provide a complete overview of qBRm used for rotavirus vaccination. We systematically searched 3 medical literature databases to identify relevant articles, in English, that were published between 2006 and 2019.

**Results:**

Of the 276 publications screened, 14 studies using qBRm for rotavirus vaccination were retained, based on preselected criteria. Four were performed in low- and middle-income countries. Almost all (13 of 14) displayed the following characteristics: force of infection assumed to be constant over time (static model), indirect effect of rotavirus vaccination (herd effect) not considered, closed model (individuals not allowed to enter and/or exit the model over time), and aggregated level (no tracking of individual’s behavior). Most of the models were probabilistic (9 of 14) and reported sensitivity and/or scenario analyses (12 of 14). Input parameter values varied across studies. Selected studies suggest that, depending on the models used, for every IS hospitalization and death induced, vaccination would prevent, respectively, 190–1624 and 71–743 RVGE-related hospitalizations and deaths.

**Conclusions:**

The benefits of rotavirus vaccination were shown to largely exceed the increased risk of IS, across all studies. Future research aiming to harmonize qBRm for rotavirus vaccination should ensure the comparability of studies and provide additional information for regulatory authorities, physicians, and patients.

Infection with rotaviruses is the most common cause of severe diarrhea and dehydration in young children. Although spread worldwide, rotavirus infection induces a higher burden in low-income countries [1]. These highly contagious viruses virtually infect all children before they reach the age of 5 [2]. Rotavirus was responsible for an estimated 258 million (95% confidence interval [CI], 193–341 million) episodes of gastroenteritis and 128 000 (95% CI, 104 500–155 600) deaths in children under the age of 5 in 2016 [3].

Historically, 9 months after an oral rhesus-human reassortant rotavirus tetravalent vaccine (RotaShield; Wyeth) was licensed in the United States in October 1998, the immunization program was suspended because of a temporal association between rotavirus vaccination and occurrence of intussusception (IS) [4]. The estimated relative risk (RR) of IS during the 3–7 days after RotaShield administration was 58.9 (95% CI, 31.7–109.6) postdose 1 and 11.0 (95% CI, 4.1–29.5) postdose 2 [5]. Intussusception is a rare but serious medical condition observed when a segment of the intestine invaginates into an adjacent distal segment [6, 7] resulting in blood vessel compression and leading to pain, bowel oedema, and—if arterial supply is compromised—intestinal ischemia, necrosis, and even perforation. If left untreated, IS can be fatal. Although rare, IS is the most common cause of acute intestinal obstruction in infants, occurring usually between 4 and 10 months of age [1]. Intussusception occurs without rotavirus vaccination with an average worldwide background incidence rate estimated at 74 cases per 100 000 children under 1 year of age, and it was shown to range between 9 and 328 per 100 000 across countries [7]. Surgical rates of IS are substantially higher in Africa (77%) and Central and South America (86%) compared with other regions (13%–50%) [7, 8].

Since 2006, 2 live-attenuated rotavirus vaccines have been licensed in more than 100 countries [9]: Rotarix (GlaxoSmithKline Biologicals), a 2-dose schedule oral human rotavirus vaccine, and RotaTeq (Merck & Co., Inc.), a 3-dose schedule oral human-bovine reassortant rotavirus vaccine [10–13]. The 2 established vaccines have proven to be effective, and they have led to a significant decline in rotavirus gastroenteritis (RVGE)-related morbidity and mortality [10, 11]. Two new rotavirus vaccines have received prequalification from the World Health Organization (WHO) in 2018: ROTAVAC (Bharat Biotech International Limited) and ROTASIIL (Serum Institute of India Limited) [14]. Moreover, other rotavirus vaccines have been licensed for national markets in China (Lanzhou Lamb rotavirus vaccine; Lanzhou Institute of Biological Products) and in Vietnam (Rotavin-M1 rotavirus vaccine; Center for Research and Production of Vaccines) [15]. In 2009, the WHO recommended rotavirus vaccination to all children, especially in countries with high diarrhea-related mortality rates [1]. By the end of 2018, 92 countries had introduced rotavirus vaccination into their routine immunization program for children [16].

Several observational postlicensure surveillance studies have been undertaken to assess the risk of IS after vaccination with Rotarix and RotaTeq in real-life settings [8, 17–24]. Data from epidemiological studies suggest that between 1 and 6 cases of IS per 100 000 vaccinated children may be attributable to rotavirus vaccination [25]. A meta-analysis has reported an overall estimate of RR of IS postdose 1 of 5.4 (95% CI, 3.9–7.4) and 5.5 (95% CI, 3.3–9.3) and postdose 2 of 1.8 (95% CI, 1.3–2.5) and 1.7 (95% CI, 1.1–2.6), after vaccination with Rotarix and RotaTeq, respectively [26]. These overall estimates were further confirmed by 2 recent meta-analyses [22, 27].

Given the increased risk of IS associated with rotavirus immunization, it is crucial to balance it with the benefits of vaccination in reducing RVGE-related hospitalizations and deaths [28, 29]. In this context, several studies have been conducted in various geographical settings to investigate the benefit-risk (BR) profile of rotavirus vaccination. These studies using quantitative BR models (qBRm) provided key information for regulatory authorities, physicians, and parents [30–33].

The aim of the present systematic literature review was as follows: (1) to provide a comprehensive overview of published qBRm focusing on rotavirus vaccination and their methodological approaches and (2) to characterize the BR profile of rotavirus vaccination on the basis of available scientific evidence.

## METHODS

### Search Strategy

We systematically searched Medline, Scopus, and the Institute for Scientific Information (ISI) Web of Knowledge databases to identify original studies on qBRm for rotavirus vaccination published from January 1, 2006 to December 13, 2019. The search strategy used prespecified terms (“benefit-risk” and “rotavirus vaccines”), as detailed in Appendix Table 1, and was limited to publications in English.

Two reviewers (H.A. and N.P.) independently screened all titles and abstracts using predefined criteria (Appendix Table 2). Subsequently, the assessment for eligibility of identified publications was carried out by examining their full text. Disagreements between the 2 reviewers were resolved through discussion. In addition, reference lists of eligible articles were screened (ie, “snowballing”) to identify potential additional publications. Finally, a gray literature search of public health organization websites and Google was performed using the prespecified search terms. All citations were downloaded and imported in EndNote (version X7; Thomson Reuters Corp., New York, NY).

### Data Extraction and Analysis

The following data were extracted and summarized: the qBRm general information, the model characteristics, the input parameters, and the BR estimates. General information includes the studied vaccine(s), the alternative(s) to the studied vaccine(s), the vaccine funding sources, and the income category of the countries for which the BR was estimated. The model characteristics were classified according to the 8 attributes [34–36] as follows:

1. Simulation versus Nonsimulation model: the BR estimates were either derived from modeling approach using simulation techniques of various degrees of complexity (eg, cohort or microsimulation models) including as many components and interaction as possible (simulation) or from a simple computation, mathematical function, or statistical model (nonsimulation).
2. Dynamic versus Static model: the force of infection was assumed to change over time (dynamic) or not (static).
3. Model considering Herd effect or Not: a potential herd effect of rotavirus vaccination was considered (yes) or not (no).
4. Model considering Waning effect or Not: a potential waning effect (ie, vaccine efficacy/effectiveness decrease with time) was considered (yes) or not (no)
5. Open versus Closed model: an open model allows individuals to enter and exit the model over time (open), whereas a closed model does not allow for new entrances over time (closed).
6. Probabilistic versus Deterministic model: the model takes into account the uncertainty around the input parameters (probabilistic) or not (deterministic).
7. Model integrating Aggregate versus Individual-based data: The population’s behavior in the model is simulated using population’s averages (aggregate data) or considering each individual’s attributes (individual-based data).
8. Model including Scenario/Sensitivity analyses or Not: scenario (using analyses to investigate different epidemiological or healthcare scenarios of interest) and/or sensitivity (using analyses to quantify the range of uncertainty) analyses were conducted or not. Sensitivity analyses were categorized between deterministic (using point estimates) and probabilistic (using probability distributions).

Input parameters used to perform qBRm along with benefit, risk, and BR ratio (BRR) after rotavirus vaccination were extracted from analyzed studies. The benefit of rotavirus vaccination was reported as the annual number or proportion of RVGE-related hospitalizations or deaths prevented by vaccination in children before 5 years of age. The risk of rotavirus vaccination was reported as the annual number or proportion of IS-related hospitalizations or deaths attributed to vaccination in children under 1 year of age. The BRR after rotavirus vaccination was expressed as the ratio of the annual number of RVGE-related hospitalizations or deaths prevented (benefit) and the annual number of IS-related hospitalizations or deaths attributed to vaccination (risk).

## RESULTS

### Study Selection

**Figure 1.**
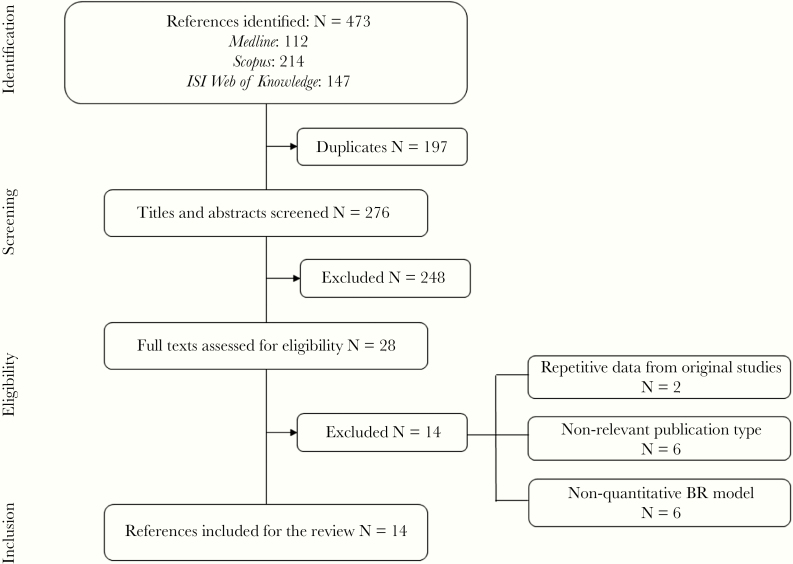
PRISMA flow diagram. ISI, Institute for Scientific Information.

After removing duplicates, the search strategy yielded 276 unique records, from which 248 were excluded based on titles and/or abstracts that were not relevant to the present analysis. The full-text review of the 28 selected articles led to the consensual exclusion of 14 of them by both reviewers, leaving 14 publications for data extraction and analysis (Figure 1).

### General Information and Model Characteristics of Selected Studies

Quantitative BR models used for rotavirus vaccination were published from 2009 onwards (Table 1) [17, 18, 37–48]. Among the 14 selected studies, 8 investigated Rotarix (6 of 14) [18, 37, 41, 44] or RotaTeq (2 of 14) [38], whereas 5 assessed both rotavirus vaccines [17, 39, 40, 42, 43]. The last study [47] investigated all currently licensed vaccines (Rotarix, RotaTeq, ROTAVAC, ROTASIIL, and RV3-BB) and were assumed to be equivalent in terms of vaccine efficacy, effectiveness, or impact, or IS risks. All studies focused on rotavirus vaccines administered according to the national or WHO recommended vaccination schedule. Five of the 14 studies also considered rotavirus vaccination without age restriction [37, 42, 43, 46, 47], and 1 study [45] considered a targeted strategy with selective rotavirus vaccination of infants with medical risk conditions (prematurity, low birth weight, or congenital conditions). Only 2 studies were reported as funded by a pharmaceutical company (GlaxoSmithKline) [41, 48], whereas the others were classified as “other sources of funding” (such as academic institutions or health authorities). Nine studies were performed in high-income countries (HICs) [17, 37, 38, 40, 41, 44–46, 48], and 5 were performed in low- and/or middle-income countries (LMICs) [18, 39, 42, 43, 47]. Studies in HICs were country-specific and mainly used local data, whereas those in LMICs were conducted across several countries: in 2, 14, 117, 135, and 158 LMICs, respectively. In the studies that included 117, 135, and 158 LMICs, a generic model using data provided by geographic area (not country-specific) was used to calculate the different estimates [42, 43, 47].

**Table 1.** General Information and Model Characteristics of Studies Using Quantitative Benefit-Risk Models for Rotavirus Vaccination

	General Information				Model Characteristics							
Source	Vaccine(s)	Alternative(s)	Funding	Income Category	Simulation Model	Static/ Dynamic Model	Herd Effect	Waning Effect	Closed / Open Model	Deterministic/ Probabilistic Model	Aggregate/ Individual- Based Model	Scenario/ Sensitivity Analyses
Patel et al [42]	Rotarix/RotaTeq	No vaccination No age restriction to vaccination	Other	LMICs	No	Static	No	No	Closed	Deterministic	Aggregate	Scenario
Patel et al [18]	Rotarix	No vaccination	Other	LMICs	No	Static	No	No	Closed	Deterministic	Aggregate	No
Desai et al [39]	Rotarix/RotaTeq	No vaccination	Other	LMICs	Yes	Static	No	No	Closed	Probabilistic	Aggregate	PSA
Patel et al [43]	Rotarix/RotaTeq	No vaccination No age restriction to vaccination	Other	LMICs	Yes	Static	No	No	Closed	Probabilistic	Aggregate	Scenario PSA
Carlin et al [17]	Rotarix/RotaTeq	No vaccination	Other	HIC	No	Static	No	No	Closed	Deterministic	Aggregate	No
Desai et al [38]	RotaTeq	No vaccination	Other	HIC	Yes	Static	No	No	Closed	Probabilistic	Aggregate	DSA/PSA
Clark et al [37]	Rotarix	No vaccination No age restriction to vaccination	Other	HIC	Yes	Static	No	Yes	Closed	Probabilistic	Aggregate	Scenario PSA
Yung et al [44]	Rotarix	No vaccination	Other	HIC	No	Static	No	No	Closed	Deterministic	Aggregate	Scenario
Ledent et al [41]	Rotarix	No vaccination	Pharma ceutical	HIC	Yes	Static	No	No	Closed	Probabilistic	Aggregate	DSA/PSA
Lamrani et al [40]	Rotarix/RotaTeq	No vaccination	Other	HIC	Yes	Static	No	Yes	Closed	Probabilistic	Aggregate	Scenario PSA
Ledent et al [48]	Rotarix	No vaccination	Pharma ceutical	HIC	Yes	Static	No	No	Closed	Probabilistic	Aggregate	DSA/PSA Scenario
Bruijning-Verhagen et al [45]	RotaTeq	No vaccination Targeted vaccination	Other	HIC	Yes	Static	Yes	Yes	Closed	Probabilistic	Aggregate	DSA/PSA Scenario
Bruun et al [46]	Rotarix	No vaccination No age restriction to vaccination	Other	HIC	No	Static	No	Yes	Closed	Deterministic	Aggregate	Scenario
Clark et al [47]	Rotarix/RotaTeq/ RV3-BB/ROTAVAC/ROTASIIL	No vaccination No age restriction to vaccination	Other	LMICs	Yes	Static	No	Yes	Closed	Probabilistic	Aggregate	PSA Scenario

Abbreviations: DSA, deterministic sensitivity analyses; HIC, high-income country; LMICs, low- and middle-income countries; PSA, probabilistic sensitivity analyses.

Studies included in the review used simulation (9 of 14) or nonsimulation models (5 of 14) to estimate final BR outcomes. All simulation models used a cohort model as modeling approach, ie, simulated a hypothetical cohort of individuals through a set of health states over time. A few studies (5 of 14) considered a waning effect over time after rotavirus vaccination. Three attributes were identical across all studies, ie, all models were static, closed, and reported results at an aggregate/population average level. Only 1 study took herd effect into account. Models were probabilistic in 9 studies and deterministic in the remaining ones. Most studies reported results from additional analyses: scenario analyses (9 of 14), probabilistic sensitivity analyses (PSA) (9 of 14), and deterministic sensitivity analyses (4 of 14). General information for each study and a description of the different models are summarized in Table 1.

### Summary of Input Parameters

Almost all (13 of 14) analyses included the following input parameters: vaccine efficacy or effectiveness (VE), vaccine coverage (VC), RR of IS after vaccination during a given risk period (Table 2), and the baseline incidence of hospitalizations or deaths (related to RVGE or IS) in children under 5 years (for RVGE) or 1 year of age (for IS) in the absence of vaccination (Table 3). One study considered baseline incidence for RVGE under <15 years [45]. Vaccine effectiveness varied according to the number of doses administered (1 to 3), the age of immunization (eg, VE >6 months and >12 months after vaccination), the vaccine used (mainly Rotarix or RotaTeq), and the health outcome of interest (hospitalization or death). Vaccine coverage considered for a full vaccination schedule was low in LMICs (approximately 50%) and high in HICs (approximately 90%). All studies considered a 7-day risk period for the risk of IS after vaccination, whereas half of them also investigated an additional risk period of up to 21 days [17, 18, 37, 40, 44, 46, 47]. The RR of IS ranged between 1.1 (95% CI, 0.3–3.3) (Brazil) and 9.9 (95% CI, 3.7–26.4) (Australia and France) after the first dose with a 7-day risk period. The RR of IS ranged from 1.7 (95% CI, 1.2–2.4) (158 LMICs assessed in the study by Patel et al [43]) to 3.1 (95% CI, 0.4–23.4) (Singapore) after the second dose with a 7-day risk period. Only 1 study analyzed a RR of IS after the third dose with a risk period of 7 days [42]. Details on RRs used according to the different risk periods are available in Table 2. The baseline incidence of hospitalizations or deaths (number and rate) for RVGE and IS in the absence of vaccination were country or area specific and were higher in LMICs for deaths (Table 3 and Appendix Table 3).

**Table 2.** Input Parameters of Quantitative Benefit-Risk Models Used for Rotavirus Vaccination

Source	Location	Vaccine(s)	Vaccine Efficacy/Effectiveness		Vaccine Coverage	IS Risk Period (Days): Relative Risk			Birth Cohort
Patel et al [42]	LMIC (117)	Rotarix/RotaTeq	D1: 50% D2 and D3: 75%		54%	D1 (1–7): 6.0			NR
						D2 (1–7): 3.0			
						D3 (1–7): 1.0			
Patel et al [18]	Brazil	Rotarix	D1 and D2: 85%		50%		Brazil	Mexico	3 068 249
						D1 (1–7):	1.1 [0.3–3.3]	5.3 [3.0–9.3]	
	Mexico	Rotarix	D1 and D2: 85%		50%				2 414 329
						D2 (1–7):	2.6 [1.3–5.2]	1.8 [0.9–3.8]	
						D1 (8–14):	1.3 [0.5–3.4]	1.1 [0.5–2.7]	
						D2 (8–14):	1.4 [0.7–3.0]	2.2 [1.1–4.2]	
						D1 (15–21):	0.2 [0.0–1.4]	0.9 [0.3–2.2]	
						D2 (15–21):	0.9 [0.4–2.0]	2.2 [1.2–4.0]	
Desai et al [39]	Latin America (14)	Rotarix/RotaTeq	Hosp: 66% [31–83] to 85% [72–93] Death: 80% [59–90] to 100% [74–100]		54%−92%	D1 (1–7): 5.3 [3.0–9.3]			9 588 000
						D2 (1–7): 2.6 [1.3–5.2]			
Patel et al [43]	LMIC (158)	Rotarix/RotaTeq	61% [44–73] to 97% [84–100]		Country-specific	D1 (1–7): 5.5 [4.1–7.5]			123 600 000
						D2 (1–7): 1.7 [1.2–2.4]			
Carlin et al [17]	Australia	Rotarix/RotaTeq	D1: 50% D2 and D3: 80%		85%		Rotarix	RotaTeq	290 446
						D1 (1–7):	6.8 [2.4–19.0]	9.9 [3.7–26.4]	
						D1 (8–21):	3.5 [1.3–8.9]	6.3 [2.8–14.4]	
						D2 (1–7):	2.8 [1.1–7.3]	2.8 [1.2–6.8]	
Desai et al [38]	United States	RotaTeq	D1: 66% [16–86] D2: 90% [75–96] D3: 92% [86–96]		D1: 96% D2: 93% D3: 82%	D1 (1–7): 5.3 [3.0–9.3]			4 261 494
Clark et al [37]	England	Rotarix	D1 >6 m: 96% [90.2–98.8] D1 >12 m: 90.7% [85.6–94.3] D2 >4 m: 100% [81.8–100] D2 >10 m: 92.2% [65.6–99.1]		D1 >15 w: 96% D2 >24 w: 94%	D1 (1–7): 6.8 [2.4–19.0]			656 457
						D1 (8–21): 3.5 [1.3–8.9]			
						D2 (1–7): 2.8 [1.1–7.3]			
						D2 (8–21): 2.1 [1.0–4.6]			
Yung et al [44]	Singapore	Rotarix	D1: 50% D2: 80%		90%	D1 (1–7): 8.4 [2.4–29.0]			40 000
						D2 (1–7): 3.1 [0.4–23.4]			
						D2 (8–21): 1.5 [0.2–11.7]			
Ledent et al [41]	Japan	Rotarix	D1: 73.9% [50.1–83.7] D2: 91.6% [62.4–99.1]		D1: 100% D2: 98% [95–98]	D1 (1–7): 5.4 [3.9–7.4]			1 018 400
						D2 (1–7): 1.8 [1.2–2.7]			
Lamrani et al [40]	France	Rotarix/RotaTeq	RotarixD1 >6 m: 96% [90.2–98.8]D1 >12 m: 90.7% [85.6–94.3]D2 >4 m: 100% [81.8–100]D2 >10 m: 92.2% [65.6–99.1]	RotaTeqD1 <12 m: 58.9% [51.7–65.0]D1 >24 m: 53.6% [46.4–59.7]D2 <12 m: 77.4% [71.1–82.1]D2 >24 m: 72.1% [65.8–76.8]D3 <12 m: 95.8% [90.5–98.2]D3 >24 m: 88.0% [82.7–90.4]	D1: 92% D2: 88% D3: 84%		Rotarix	RotaTeq	765 550
						D1 (1–7):	6.8 [2.4–19.0]	9.9 [3.7–26.4]	
						D1 (8–21):	3.5 [1.3–8.9]	6.3 [2.8–14.4]	
						D2 (1–7):	2.8 [1.1–7.3]	2.8 [1.2–6.8]	
						D2 (8–21):	2.1 [1.0–4.6]	1.8 [0.8–3.9]	
Ledent et al [48]	France	Rotarix	D1: 75% [55–88] D2: 90% [81–95]		D1: 100% D2: 92% [72–100]	D1 (1–7): 5.4 [3.9–7.4]			791 183
						D2 (1–7): 1.8 [1.3–2,5]			
Bruijning-Verhagen et al [45]	Netherlands	RotaTeq	D1 and D2: 88% D3: 94.8%		86%	NR			171 387
Bruun et al [46]	Norway	Rotarix	D1 and D2: 93% [87–98]		D1: 91% D2: 86%	D1 (1–21): 2.4 [1.5–3.8]			60 000
						D2 (1–21): 1.8 [1.3–2.4]			
Clark et al [47]	LMIC (135)	Rotarix/RotaTeq/ ROTAVAC/ROTASIIL/ RV3-BB	79% [75–82] to 100% [99–100]		Country-specific	D1 (1–7): 6.3 [4.3–9.2]			60 000 000
						D1 (8–21): 1.7 [1.1–2.7]			
						D2 (1–7): 1.8 [1.4–2.3]			
						D2 (8–21): 1.4 [1.0–1.8]			

Abbreviations: D1, dose 1; D2, dose 2; D3, dose 3; Hosp, hospitalization; IS, intussusception; LMICs, low- and middle-income countries; m, months; N, number; NR, not reported; w, weeks.

**Table 3.** Benefit-Risk Estimates of Rotavirus Vaccination in Analyzed Studies

Source	Location	Vaccine(s)	Events	Baseline Incidence RVGE <5 y (N)	Prevented RVGE <5 y (N)	Prevented RVGE <5 y (%)	Baseline Incidence IS <1 y (N)	Caused IS <1 y (N)	Caused IS <1 y (%)	BRR (RVGE/IS)
Patel et al [42]	LMIC (117)	Rotarix/RotaTeq	Hosp	NR	NR	NR	NR	NR	NR	NR
			Death	517 959	194 564	37.6^a^	NR	1106	NR	176^a^
Patel et al [18]	Brazil	Rotarix	Hosp	92 453	69 572	75.3^a^	2146	55	2.6^a^	1265^a^
			Death	850	640	75.3^a^	107	3	2.8^a^	213^a^
	Mexico	Rotarix	Hosp	16 086	11 551	71.8^a^	1215	41	3.4^a^	282^a^
			Death	923	663	71.8^a^	61	2	3.3^a^	332^a^
Desai et al [39]	Latin America (14)	Rotarix/RotaTeq	Hosp	229 656	144 746 [128 821–156 707]^c^	63.0^a^	5556	172 [126–293]^c^	3.1^a^	841 [479–1142]^c^
			Death	6302	4124 [3740–4239]^c^	65.4^a^	326	10 [6–17]^c^	3.1^a^	395 [207–526]^c^
Patel et al [43]	LMIC (158)	Rotarix/RotaTeq	Hosp	NR	NR	NR	NR	NR	NR	NR
			Death	452 800 [386 600–519 900]^a,b^	155 800 [83 300–217 700]^b^	34.4^a^	NR	253 [76–689]^b^	NR	615^a^
Carlin et al [17]	Australia	Rotarix/RotaTeq	Hosp	11 073	6528	59.0^a^	144	14	9.7^a^	466^a^
			Death	NR	NR	NR	NR	NR	NR	NR
Desai et al [38]	United States	RotaTeq	Hosp	71 175 [50 131–96 802]^b^	53 444 [37 622–72 882]^b^	75.1^a^	NR	45 [21–86]^b^	NR	1093 [688–1902]^b^
			Death	33 [23–43]^b^	14 [10–19]^b^	42.4^a^	NR	0.2 [0.1–0.3]^b^	NR	71 [48–112]^b^
Clark et al [37]	England	Rotarix	Hosp	14 770 [14 113–15 427]^a,b^	13 276 [12 255–14 181]^b^	89.9^a^	248	35.4 [7.0–97.6]^b,d^	14.3^a^	375 [136–1900]^b,d^
			Death	3.3 [1.7–4.9]^b^	2.9 [1.7–4.1]^b^	86.7^a^	0.3^a^	0.1 [0.0–0.2]^b,d^	10.6^a^	88 [18–852]^b,d^
Yung et al [44]	Singapore	Rotarix	Hosp	808	570	70.5^a^	22	3	13.6^a^	190^a^
			Death	NR	NR	NR	NR	NR	NR	NR
Ledent et al [41]	Japan	Rotarix	Hosp	20 829 [16 301–26 129]^b^	17 925 [11 715–23 276]^b^	86.1^a^	1571 [1308–1868]^b^	50 [7.2–237]^b^	3.2^a^	350 [69–2510]^b^
			Death	7.3 [5.7–9.3]^b^	6.3 [4.1–8.2]^b^	86.3^a^	0.5 [0.2–1.2]^b^	0.1 [0.0–0.1]^b^	3.1^a^	366 [59–3271]^b^
Lamrani et al [40]	France	Rotarix/RotaTeq	Hosp	11 866^a^	10 375 [7802–13 293]^a^	87.4^a^	214	47 [25–81]^b^	21.9^a^	214 [128–362]^b^
			Death	16 [15–18]	14 [12–15]^a^	87.5^a^	0.3	0.1 [0.0–0.2]^b^	17.8^a^	273 [89–1228]^b^
Ledent et al [48]	France	Rotarix	Hosp	15 059 [12 100–18 476]^b^	11 132 [7841–14 409]^b^	73.9^a^	323 [257–400]^b^	6.9 [2.3–38.4]^b^	2.1^a^	1624 [240–5243]^b^
			Death	10.1 [4.6–19.5]^b^	7.43 [3.27–14.68]^b^	73.3^a^	0.5 [0.2–0.9]^b^	0.1 [0.0–0.1]^b^	2.2^a^	743 [93–3723]^b^
Bruijning-Verhagen et al [45]	Netherlands	RotaTeq	Hosp	2700 [2400–3000]^a,e^	2000 [1800–2200]^a,e^	74.1^a,e^	NR	2.9^a^	NR	685 [603–767]^e^
			Death	5.5 [3.0–8.8]^a,e^	5.2 [2.8–8.3]^a,e^	93.6^a,e^	NR	NR	NR	NR
Bruun et al [46]	Norway	Rotarix	Hosp	NR	1768 [1761–1774]^a,b^	NR	22 [19–26]^a^	1 [1–2]^b^	5.7^a^	1360
			Death	NR	NR	NR	NR	NR	NR	NR
Clark et al [47]	LMIC (135)	Rotarix/RotaTeq/ ROTAVAC/ ROTASIIL/RV3-BB	Hosp	NR	NR	NR	NR	NR	NR	NR
			Death	194 471 [158 603–257 080]^b^	62 485 [47 895–83 238]^b^	32.1^a^	NR	122 [44–322]^b^	NR	512 [218–1338]^b^

Abbreviations: BRR, benefit-risk ratio; Hosp, hospitalization; IS, intussusception; LMIC, low- and middle-income countries; N, number; NR, not reported; RVGE, rotavirus gastroenteritis; y, years.

^a^Using data from original publications.

^b^Median values.

^c^90% CI.

^d^IS risk period (0–2 years).

^e^Baseline incidence RVGE <15 years.

### Benefit-Risk Estimates of Rotavirus Vaccination

**Figure 2.**
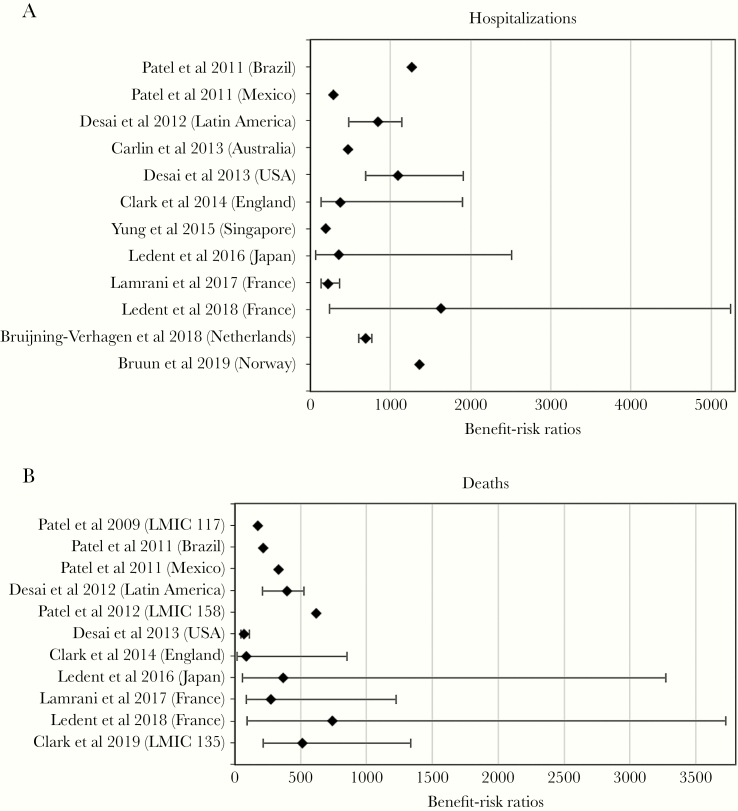
Forest plot of benefit-risk ratios associated with rotavirus vaccination from selected studies. (a) Hospitalizations. (b) Deaths. Confidence intervals were not reported for all modeling studies.

Based on the 14 selected publications, vaccination would prevent 59.0% (Australia) to 89.9% (England) of RVGE-related hospitalizations and 32.1% (mean percentage in 135 LMICs assessed by Clark et al [47]) to 87.5% (Lamrani et al [40] in France) of RVGE deaths expected to occur in a no-vaccination scenario in children under 5 years of age. On the other hand, the IS-related hospitalization rate and the IS-related death rate would increase by 2.1% (Ledent et al [48] in France) to 21.9% (Lamrani et al [40] in France) and 2.2% (Ledent et al [48] in France) to 17.8% (Lamrani et al [40] in France) as a result of vaccination in children under 1 year of age, respectively. Benefit-risk ratios ranged from 190 (Singapore) to 1624 (Ledent et al [48] in France) RVGE-related hospitalizations prevented for every additional vaccine-related IS hospitalization, whereas 71 (United States) to 743 (Ledent et al [48] in France) RVGE-related deaths would be prevented for every additional IS-death caused by the vaccine (Table 3 and Figure 2).

## DISCUSSION

To our knowledge, the present review is the first to gather available evidence on qBRm used for rotavirus vaccination. It had 2 main objectives: (1) to describe methodological approaches used in the selected models (ie, their model characteristics and input parameters) and (2) to characterize the BR profile of rotavirus vaccination based on the available scientific evidence.

Although a herd effect has been observed for rotavirus vaccination [49–54], only 1 study considered it as a model characteristic, among the 14 selected studies. Some authors argued that this choice intended to make the approach more conservative. In addition, more complex modeling techniques such as transmission dynamic models were not used at all, and PSA were only conducted in 9 of 14 of the qBRm. Choosing for simpler approaches might be explained by the fact that some studies were not conducting qBRm as primary but as secondary objective. Although 5 studies considered a waning effect of rotavirus vaccination [37, 40, 45–47], their estimates of the proportion of RVGE-related hospitalizations or deaths prevented by vaccination in children aged less than 5 years were similar to figures reported in the other studies. This might be explained by the fact that the majority of severe RVGE cases occurs during infancy [2], before the protective effect of vaccination starts to wane.

Comparing input parameters across the different studies showed that lower VC figures were considered in LMIC than in HIC studies. This might be linked to the year of publication, ie, between 2009 and 2012, the WHO-recommended administration of the first dose of rotavirus vaccines with an upper age limit of 12–14 weeks to minimize the potential risk of IS. This strict age restriction may have reduced VC in some developing countries where the timeliness of pediatric vaccination varies widely [55]. In 2013, the WHO removed this age restriction to improve VC [56]. However, it is worth noting that the use of different VC figures had no impact on BRR estimates, because none of the selected qBRm considered transmission dynamic modeling.

The annual number of IS-related hospitalizations or deaths in children less than 1 year of age used as input parameter also varied across studies. The etiology of IS is not yet clearly understood. Differences in infant diet, breastfeeding, maternal antibody levels, and association with several pathogens including adenoviruses might all contribute to the variances in background rates of IS [7, 57, 58]. A higher number of IS-related deaths are observed in LMICs compared with HICs among selected studies. This finding might be due to differences in healthcare infrastructure or delays in care [7].

The present review systematically collected the published information on qBRm for rotavirus vaccination, which allowed further characterizing its BR profile. All selected studies concluded that vaccine-prevented RVGE-related hospitalizations and deaths outweigh vaccine-induced IS-related hospitalizations and deaths, with no marked difference between LMICs and HICs. Differences in BRR noted across studies included in this review can be explained by (1) the choice of model attributes (eg, simulation versus nonsimulation models), (2) varying epidemiology of RVGE and IS observed across countries and areas, (3) data availability at the time of the study, and (4) differences in the choice of input parameter values. Nevertheless, it is crucial to consider those differences when comparing models and their outputs. For example, despite the fact that they used similar modeling approaches, the 2 qBRm studies conducted in France showed differences with BRRs of 214 and 273 in Lamrani et al ([40]) and 1624 and 743 in Ledent et al ([48]), for hospitalizations and deaths, respectively. This might be explained by the value of some input parameters (eg, risk period duration and RR of IS). In this specific example, the use of scenario analysis by Ledent [48] et al considering the same risk period of 21 days as Lamrani et al [40] resulted in similar BRR between both studies [48].

This systematic literature review has some limitations. First, the search strategy may have not identified all relevant studies, notably due to the lack of limited specific keywords for qBRm. In addition, some studies conducted by or for local governments or pharmaceutical companies may not have been made publicly available or indexed. Second, the data from included studies were not pooled in a meta-analysis to estimate an overall BRR for rotavirus vaccination, because 95% CIs were not available for all studies. Nevertheless, a forest plot allowing a visual assessment of differences between BRRs is depicted without providing overall BRR estimate (Figure 2).

## CONCLUSIONS

The present review provides a comprehensive overview of publications reporting on qBRm for rotavirus vaccination. This evidence confirms the favorable benefit-risk profile of rotavirus vaccines. The observed differences in qBRm approaches between studies complexified the comparison of their outputs and warrant the need for harmonization in such analysis to ensure comparability. In addition, because most studies focused on HICs, there is a need to increase BRR estimations in LMICs considering setting-specific input parameters and including sensitivity and/or scenario analyses to fully capture their effect.

## Supplementary Data

Supplementary materials are available at *Open Forum Infectious Diseases* online. Consisting of data provided by the authors to benefit the reader, the posted materials are not copyedited and are the sole responsibility of the authors, so questions or comments should be addressed to the corresponding author.

ofaa087_suppl_Supplementary_Tables
