# Supplementary material for: Quantitative Benefit-Risk Models Used for Rotavirus Vaccination: A Systematic Review
Source: Open Forum Infect Dis. 2020 Mar 12;7(4):ofaa087. doi: 10.1093/ofid/ofaa087 (PMC7148003; doi:10.1093/ofid/ofaa087)
Supplement: ofaa087_suppl_Supplementary_Tables [file ofaa087_suppl_supplementary_tables.docx]

**Supplemental Material on Material and Methods and Results**

**Quantitative benefit-risk models used for rotavirus vaccination: a systematic review**

**Appendix Table 1.** Research strategy and number of publications retrieved at each step in the three different databases.

**Appendix Table 2.** Exclusion criteria for the systematic review of quantitative benefit-risk models of vaccines.

**Appendix Table 3.** Estimates of the burden of disease attributable to intussusception and rotavirus in the absence of rotavirus vaccination

| **Database** | **Research strategy** | **Number of publications** |
| --- | --- | --- |
| **MEDLINE** | ((((((("benefit" [TIAB] AND "risk" [TIAB]) OR ("benefits" [TIAB] AND "risks" [TIAB]) OR ("benefits" [TIAB] AND "risk" [TIAB]) OR ("benefit" [TIAB] AND "risks" [TIAB])))) AND (("rotavirus" [TIAB] and ((“vaccines” [Mesh]) OR (“vaccine” [TIAB] OR “vaccines” [TIAB])))))) Filters: Publication date from 2006/01/01 to 2019/12/13; English | **112** |
| **SCOPUS** | ( TITLE-ABS-KEY ( ( ( "benefit" AND "risk" ) OR ( "benefits" AND "risks" ) OR ( "benefits" AND "risk" ) OR ( "benefit" AND "risks" ) ) ) ) AND ( TITLE-ABS-KEY ( ( "rotavirus" AND ( "vaccine" OR "vaccines" ) ) ) ) AND ( LIMIT-TO ( SUBJAREA , "MEDI " ) OR LIMIT-TO ( SUBJAREA , "IMMU " ) OR LIMIT-TO ( SUBJAREA , "PHAR " ) OR LIMIT-TO ( SUBJAREA , "NURS " ) OR LIMIT-TO ( SUBJAREA , "HEAL " ) OR LIMIT-TO ( SUBJAREA , "NEUR " ) OR LIMIT-TO ( SUBJAREA , "CENG " ) OR LIMIT-TO ( SUBJAREA , "MULT " ) OR LIMIT-TO ( SUBJAREA , "MATH " ) OR LIMIT-TO ( SUBJAREA , "PSYC " ) OR LIMIT-TO ( SUBJAREA , "DECI " ) OR LIMIT-TO ( SUBJAREA , "ECON " ) OR LIMIT-TO ( SUBJAREA , "COMP " ) OR LIMIT-TO ( SUBJAREA , "CHEM " ) OR LIMIT-TO ( SUBJAREA , "MATE " ) OR LIMIT-TO ( SUBJAREA , "Undefined " ) ) AND ( EXCLUDE ( DOCTYPE , "no " ) OR EXCLUDE ( DOCTYPE , "ed " ) OR EXCLUDE ( DOCTYPE , "le " ) ) AND ( LIMIT-TO ( LANGUAGE , "English " ) ) AND ( LIMIT-TO ( PUBYEAR , 2019 ) OR LIMIT-TO ( PUBYEAR , 2018 ) OR LIMIT-TO ( PUBYEAR , 2017 ) OR LIMIT-TO ( PUBYEAR , 2016 ) OR LIMIT-TO ( PUBYEAR , 2015 ) OR LIMIT-TO ( PUBYEAR , 2014 ) OR LIMIT-TO ( PUBYEAR , 2013 ) OR LIMIT-TO ( PUBYEAR , 2012 ) OR LIMIT-TO ( PUBYEAR , 2011 ) OR LIMIT-TO ( PUBYEAR , 2010 ) OR LIMIT-TO ( PUBYEAR , 2009 ) OR LIMIT-TO ( PUBYEAR , 2008 ) OR LIMIT-TO ( PUBYEAR , 2007 ) OR LIMIT-TO ( PUBYEAR , 2006 ) ) AND ( LIMIT-TO ( LANGUAGE , "English" ) ) | **214** |
| **ISI Web of Science** | TOPIC: (((("benefit" AND "risk") OR ("benefits" AND "risks") OR ("benefits" AND "risk") OR ("benefit" AND "risks")))) AND TOPIC: (("rotavirus" AND (“vaccine” OR “vaccines”))) Refined by: LANGUAGES: ( ENGLISH ) Timespan: 2006-2019. Indexes: SCI-EXPANDED, SSCI, A&HCI, CPCI-S, CPCI-SSH, BKCI-S, BKCI-SSH, ESCI, CCR-EXPANDED, IC. | **147** |

**Appendix Table 1.** Research strategy and number of publications retrieved at each step in the three different databases.

ISI – Institute for Scientific Information

**Appendix Table 2.** Exclusion criteria for the systematic review of quantitative benefit-risk models of vaccines.

| **Exclusion criteria** |
| --- |
| - The following research question is not addressed: Original studies using quantitative benefit-risk models for rotavirus vaccination; - Articles that describe non-relevant publication types (e.g. letters to the editor, editorials or comments, clinical practice guidelines, electronic textbooks); - Repetitive data from original studies; - Genetic, biochemistry, molecular or animal studies; - Economic evaluation (e.g. cost-effectiveness, cost-utility or cost-benefit studies); - Unable to access the full-text; - Publications not written in English. |

**Appendix Table 3.** Estimates of the burden of disease attributable to intussusception and rotavirus in the absence of rotavirus vaccination

| **Income**  **category** | **Source** | **Location** | **Birth cohort** | **Events** | **Baseline incidence**  **RVGE<5year (N)** | **Baseline incidence**  **RVGE<5year (Rate per 100,000)** | **Baseline incidence**  **IS <1year (N)** | **Baseline incidence**  **IS <1year (Rate per 100,000)** |
| --- | --- | --- | --- | --- | --- | --- | --- | --- |
| **LMIC** | Patel MM, et al. 2009 | LMIC (117) | NR | ‘Hosp’ | NR | NR | NR | NR |
|  |  |  |  | Death | 517,959 | NR | NR | NR |
|  | Patel MM, et al. 2011 | Brazil | 3,068,249 | ‘Hosp’ | 92,453 | 603^a^ | 2,146 | 70^a^ |
|  |  |  |  | Death | 850 | 5.5^a^ | 107 | 3.5^a^ |
|  |  | Mexico | 2,414,329 | ‘Hosp’ | 16,086 | 133^a^ | 1,215 | 50^a^ |
|  |  |  |  | Death | 923 | 7.7^a^ | 61 | 2.5^a^ |
|  | Desai R, et  al. 2012 | Latin  America (14) | 9,588,000 | ‘Hosp’ | 229,656 | 479^a^ | 5,556 | 58^a^ |
|  |  |  |  | Death | 6,302 | 13.2^a^ | 326 | 3.4^a^ |
|  | Patel MM, et al. 2012 | LMIC (158) | 123,600,000 | ‘Hosp’ | NR | NR | NR | NR |
|  |  |  |  | Death | 452,800 [386,600;519,900]^a, b^ | 73.3^a^ | NR | NR |
|  | Clark A, et  al. 2019 | LMIC (135) | 60,000,000 | ‘Hosp’ | NR | NR | NR | NR |
|  |  |  |  | Death | 194,471 [158,603;257,080]^b^ | 64.8^a^ | NR | NR |
| **HIC** | Carlin JB, et  al. 2013 | Australia | 290,446 | ‘Hosp’ | 11,073 | 762^a^ | 144 | 50^a^ |
|  |  |  |  | Death | NR | NR | NR | NR |
|  | Desai R, et  al. 2013 | US | 4,261,494 | ‘Hosp’ | 71,175 [50,131;96,802]^b^ | 334^a^ | NR | NR |
|  |  |  |  | Death | 33 [23;43]^b^ | 0.2^a^ | NR | NR |
|  | Clark A, et  al. 2014 | England | 656,457 | ‘Hosp’ | 14,770 [14,113;15,427]^a, b^ | 450^a^ | 248 | 38^a^ |
|  |  |  |  | Death | 3.3 [1.7;4.9]^b^ | 0.1^a^ | 0.3^a^ | 0.1^a^ |
|  | Yung CF, et  al. 2015 | Singapore | 40,000 | ‘Hosp’ | 808 | 404^a^ | 22 | 55^a^ |
|  |  |  |  | Death | NR | NR | NR | NR |
|  | Ledent E, et  al. 2016 | Japan | 1,018,400 | ‘Hosp’ | 20,829 [16,301;26,129]^b^ | 409^a^ | 1,571 [1,308;1,868]^b^ | 154^a^ |
|  |  |  |  | Death | 7.3 [5.7;9.3]^b^ | 0.1^a^ | 0.5 [0.2;1.2]^b^ | 0.1^a^ |
|  | Lamrani A, et al. 2017 | France | 765,550 | ‘Hosp’ | 11,866^a^ | 310^a^ | 214 | 28^a^ |
|  |  |  |  | Death | 16 [15;18] | 0.4^a^ | 0.3 | 0.1^a^ |
|  | Ledent E, et  al. 2018 | France | 791,183 | ‘Hosp’ | 15,059 [12,100;18,476]^b^ | 381^a^ | 323 [257;400]^b^ | 41^a^ |
|  |  |  |  | Death | 10.1 [4.6;19.5]^b^ | 0.3^a^ | 0.5 [0.2;0.9]^b^ | 0.1^a^ |
|  | Bruijning-Verhagen P, et al. 2018 | Netherlands | 171,387 | ‘Hosp’ | 2,700 [2,400;3,000]^a, d^ | 105^a^ | NR | NR |
|  |  |  |  | Death | 5.5 [3.0;8.8]^a, d^ | 0.2^a^ | NR | NR |
|  | Bruun T, et  al. 2019 | Norway | 60,000 | ‘Hosp’ | NR | NR | 22.3 [18.7;26.3]^a^ | 37^a^ |
|  |  |  |  | Death | NR | NR | NR | NR |

LMIC, Low-middle-income countries; HIC, High-income country; ‘Hosp’, Hospitalisation; RVGE, Rotavirus Gastroenteritis; IS, Intussusception; Mean [95% Confidence Interval (CI)]; NR, Not reported; N, Number; ^a^calculated using data from original publications; ^b^median values; ^c^90% CI; ^d^baseline incidence RVGE <15years.
